# Carotid Revascularization Improves Balance and Mobility, Particularly in Patients That Are Most Impaired

**DOI:** 10.1093/geroni/igaa057.387

**Published:** 2020-12-16

**Authors:** Vicki Gray, Sarasijhaa Desikan, Amir Khan, Dawn Barth, Siddhartha Sikdar, John Sorkin, Brajesh Lal

**Affiliations:** 1 University of Maryland School of Medicine, Baltimore, Maryland, United States; 2 George Mason University, Fairfax, Virginia, United States; 3 University of Maryland School of Medicine, Balitmore, Maryland, United States; 4 University of Maryland, Baltimroe, Maryland, United States

## Abstract

Balance and mobility function worsen with age, and more so for those with underlying diseases. Our research has demonstrated that asymptomatic carotid artery stenosis (ACAS) is associated with worse balance and mobility, and a higher fall risk, compared to older adults with similar comorbidities, but without ACAS. Thus, ACAS, with attendant blood flow-restriction to the brain is a potentially modifiable risk factor for balance and mobility dysfunction. The purpose of this study was to evaluate the impact of restoring blood flow to the brain by carotid revascularization, on balance and mobility in patients with high-grade ACAS (≥70% diameter-reducing stenosis). Twenty adults (67.0±9.4 years) undergoing carotid revascularization for high-grade stenosis were enrolled. A balance and mobility assessment was performed before- and six weeks- after carotid revascularization and included: Short Physical Performance Battery (SPPB), Berg Balance Scale (BBS), Four Square Step Test (FSST), Dynamic Gait Index (DGI) Timed Up and Go (TUG), gait speed, MiniBESTest, and Walk While Talk (WWT) test. Paired t-tests assessed changes in outcome measures between the two-time points. Significant improvements were observed in measures that combined walking with dynamic movements, DGI (P=0.003), and MiniBESTest (P=0.021). Pearson’s correlations examined the relationship between balance and mobility before surgery and change score after surgery. Patients with lower baseline DGI and MiniBest scores demonstrated the most improvement on follow-up testing (r=-0.70, p=0.001, and r=-0.59, p=0.006, respectively). In conclusion, revascularization of a carotid artery stenosis improves balance and mobility; the greatest improvements are observed in those patients that are the most impaired.

